# Polymerizable Skin Hydrogel for Full Thickness Wound Healing

**DOI:** 10.3390/ijms23094837

**Published:** 2022-04-27

**Authors:** Mairobi Persinal-Medina, Sara Llames, Manuel Chacón, Natalia Vázquez, Marta Pevida, Ignacio Alcalde, Sergio Alonso-Alonso, Laura María Martínez-López, Jesús Merayo-Lloves, Álvaro Meana

**Affiliations:** 1 Instituto Universitario Fernández-Vega, Fundación de Investigación Oftalmológica, Universidad de Oviedo, 33012 Oviedo, Spain; mairobimedina@gmail.com (M.P.-M.); m.chacon@fio.as (M.C.); natalia.vazquez@fio.as (N.V.); marta.pevida@cruzroja.es (M.P.); nacho.alcalde@fio.as (I.A.); sergio.alonso@fio.as (S.A.-A.); merayo@fio.as (J.M.-L.); meana@fio.as (Á.M.); 2 Instituto de Investigación Sanitaria del Principado de Asturias (ISPA), 33011 Oviedo, Spain; 3 Centro de Investigación Biomédica en Red en Enfermedades Raras (CIBERER) ISCIII, 28029 Madrid, Spain; 4 Unidad de Ingeniería Tisular, Centro Comunitario Sangre y Tejidos de Asturias (CCST), 33006 Oviedo, Spain; 5 Instituto de Investigación Sanitaria-Fundación Jiménez Díaz (IIS-FJD), 28015 Madrid, Spain; 6 Instituto Oftalmológico Fernández-Vega, 33012 Oviedo, Spain; laura.martinez@fernandez-vega.com

**Keywords:** skin regeneration, tissue engineering, cellular therapy, hydrogel

## Abstract

The skin is the largest organ in the human body, comprising the main barrier against the environment. When the skin loses its integrity, it is critical to replace it to prevent water loss and the proliferation of opportunistic infections. For more than 40 years, tissue-engineered skin grafts have been based on the in vitro culture of keratinocytes over different scaffolds, requiring between 3 to 4 weeks of tissue culture before being used clinically. In this study, we describe the development of a polymerizable skin hydrogel consisting of keratinocytes and fibroblast entrapped within a fibrin scaffold. We histologically characterized the construct and evaluated its use on an in vivo wound healing model of skin damage. Our results indicate that the proposed methodology can be used to effectively regenerate skin wounds, avoiding the secondary in vitro culture steps and thus, shortening the time needed until transplantation in comparison with other bilayer skin models. This is achievable due to the instant polymerization of the keratinocytes and fibroblast combination that allows a direct application on the wound. We suggest that the polymerizable skin hydrogel is an inexpensive, easy and rapid treatment that could be transferred into clinical practice in order to improve the treatment of skin wounds.

## 1. Introduction

The skin is the largest organ of the body accounting for about 15% of the total body weight in adults [1]. Skin performs many vital functions including protection (UV light absorption, pathogens, mechanical), perception (touch, temperature, pain) and regulation (thermal, hydration, excretion) [2]. Skin injuries caused by burns, chronic ulcers and genetic or somatic diseases cause loss of skin integrity that compromises the skin barrier and impairs its physiological functions; requiring effective treatment to prevent morbidity and mortality [3]. In cases where a significant amount of skin tissue is lost due to injury, it is essential to replace it to prevent water loss and to mitigate the risk of opportunistic pathogens that could lead to infections. The World Health Organization (Geneva, Switzerland) estimates that nearly 11 million burn injuries per year worldwide require medical attention, with approximately 180,000 leading to death [4,5].

To restore damaged skin, different skin models developed by tissue engineering are used [6]. Since keratinocytes were successfully cultured in the laboratory 40 years ago [7], the transplant of autologous keratinocyte sheets became a relatively common clinical practice, however it still presents many limitations that prevent its worldwide adoption: fragility, difficult handling, unpredictable take rate, high cost and sensitivity to mechanical forces due to the lack of a dermal component [8,9]. In the following years, the development of bilayer skin substitutes was attempted in order to mitigate these limitations. Each bilayer skin substitute model has its own characteristics but all of them have in common a first epithelial layer and a second dermal layer below it. The dermal component of these substitutes can be composed of synthetic substrates [6], acellular protein matrix [10,11], decellularized dermis [12] or fibroblasts dispersed within protein scaffold [13]. In the last case, fibrin, a component derived from blood plasma, has been widely used as a scaffold to develop human dermis equivalents, having the advantages of low price, high availability and good tolerance to keratinocytes and fibroblasts [14,15]. Additionally, fibrin can be produced as an autologous scaffold [16]. However, these bilayer approaches require incubation periods ranging from 3 to 4 weeks to form a fully differentiated epithelium prior to transplant.

Guided by our experience producing skin equivalents based on plasma for human transplantation [17,18,19,20,21], we attempt to produce a completely autologous polymerizable skin that can be fabricated directly onto the wound bed. Moreover, the thickness of this hydrogel does not limit its nutrition from the vascular network present in the wound bed.

Polymerizable hydrogels are gaining much recent attention due to their soft properties similar to extracellular matrix, adjustable physical and chemical properties, and their ability to fill any irregular shape wound [22]. They can keep wounds moist, absorb excess tissue exudate, allow gas permeation, rehydrate eschar, aid in autolytic debridement, protect wounds against infection, scavenge free radicals [23] and can act as a reservoir for therapeutic agents or other chemical substances [24,25].

In this study, we have developed a two-component hydrogel, consisting of thrombin concentrate (polymerization-inducing agent) and fibrinogen (clotting component). This system allows the hydrogel to be stably deposited on the wound bed and to instantaneously polymerize. Here, we describe our proof-of-concept study to engineer human skin in a layer-by-layer assembly process using fibroblasts and keratinocytes as representative cell types. We proved that this polymerizable skin hydrogel can be applied directly onto the wound, avoiding the previous culturing and differentiation processes of conventional skin equivalents, and decreasing the cost-effectiveness ratio by reducing the time of skin production. We present a novel fully autologous approach in which all its components could be easily obtained from the patient itself. We show the outcome of the in vitro results of this new model of polymerizable human skin hydrogel, as well as a histological study of the wound healing animal model treated with this hydrogel.

## 2. Results

### 2.1. Analysis of Thrombin Concentrate and Fibrinogen Samples

Thrombin activity and fibrinogen concentration were quantified. The stability of these two parameters after freezing and thawing at different times was also studied.

**Figure 1:**
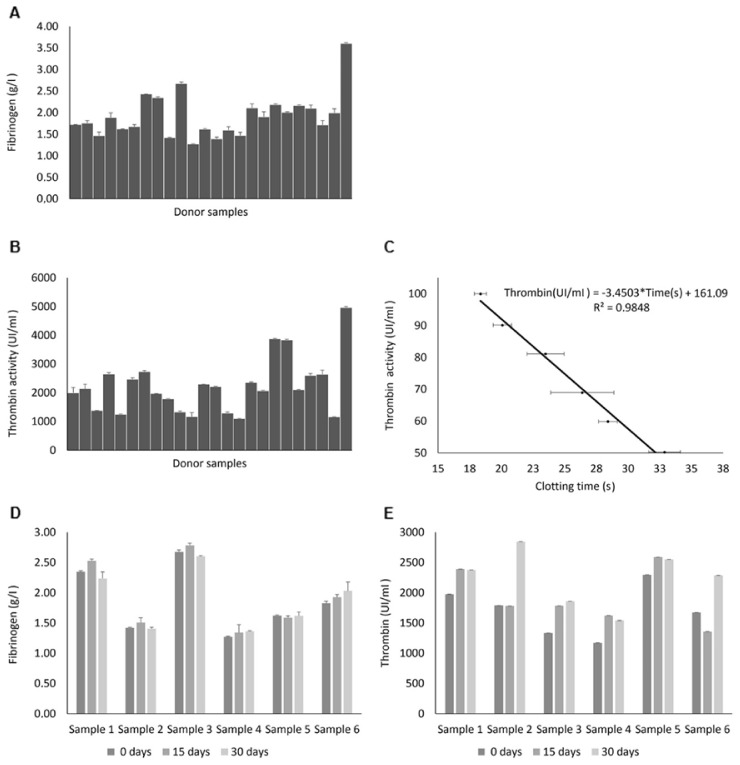
Analysis of thrombin concentrate and fibrinogen samples. (**A**) Fibrinogen concentration (g/L) in each FFP used. (**B**) Enzymatic activity (UI/mL) in each thrombin concentrate produced. (**C**) Standard curve used to calculate the enzymatic activity of each thrombin concentrate [y (UI/mL) = −3.4503 × x(s) + 161.09]. (**D**) Fibrinogen concentration (g/L) variation after storage at −80 °C and thawing at different times up to 30 days. Samples did not show a significative depletion (*p* > 0.05). (**E**) Enzymatic activity (UI/mL) variation after storage at −80 °C and thawing at different times up to 30 days. Samples did not show a significative depletion (*p* > 0.05). Data expressed as mean ± SD.

The average concentration of fibrinogen in the FFPs was 1.92 ± 0.51 g/L. The average enzymatic activity in the thrombin concentrate was 2225.02 ± 952.00 UI/mL, and the pH was kept close to 7.0 in all cases. Thrombin concentrate activity and fibrinogen concentration did not show any significantly statistical difference after freeze storage at −80 °C up to 30 days (Figure 1).

### 2.2. Viability Assay

Thrombin concentrate and fibrinogen toxicities were studied in fibroblasts.

Neither thrombin concentrate nor fibrinogen significantly reduced cell viability in fibroblasts after 24 h exposure (Control: 98.16 ± 2.03%; Thrombin concentrate: 83.93 ± 21.43%; Fibrinogen: 98.64 ± 1.36%). Moreover, there was no statistical difference between cell densities after the exposure period between any group (Control: 271 ± 27 cells/cm^2^; Thrombin concentrate: 242 ± 29 cells/cm^2^; Fibrinogen: 232 ± 20 cells/cm^2^) (Table 1).

### 2.3. Polymerizable Hydrogel Formation

The fibrinogen could not form fibrin hydrogels in the absence of thrombin. The time of polymerization was less than the sensitivity of the fibrin timer (≤5 s) after mixing fibrinogen with the thrombin concentrate.

### 2.4. In Vitro Fabrication of Polymerizable Skin Hydrogel

Both skin hydrogel polymerization and cell growth were analyzed in vitro.

Polymerizable skin hydrogels fabricated with thrombin concentrate were not homogeneous. The rapid speed of polymerization caused bulges on the surface of the gels. So thrombin concentrate diluted 1:10 in calcium buffer, was used for the following experiments. Polymerizable skin hydrogels fabricated with diluted thrombin concentrate, were homogeneous, attached to the transwell membrane and did not retract during the culture period or during the air-liquid interface phase.

**Figure 2:**
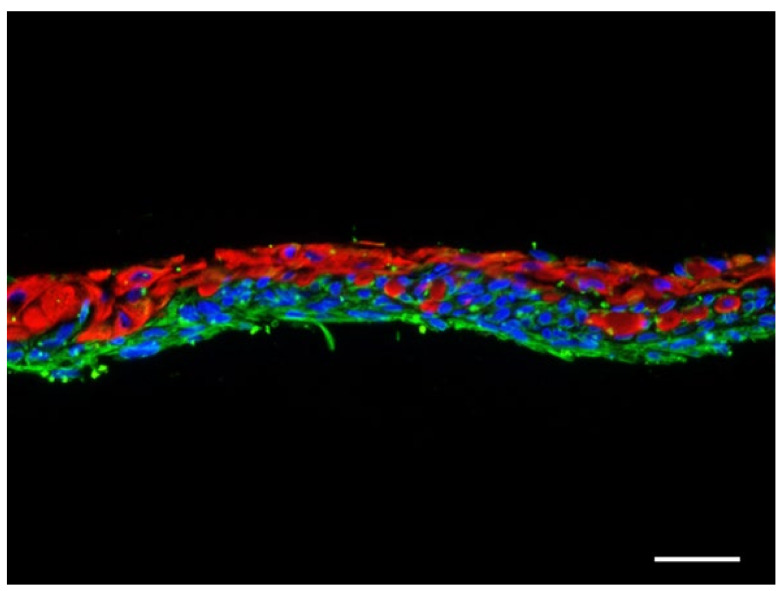
Immunofluorescence study of skin hydrogels fabricated in vitro after 7 days of air-liquid culture. Continuous keratinocyte layer (in red, excitation/emission: 480/527, labelled with anti-human high molecular weight cytokeratin antibody) firmly attached to the underlying fibroblasts-containing hydrogel. Fibroblasts (in green, excitation/emission: 360/470, labelled with anti-vimentin antibody) grew homogeneously across the dermal layer. Scale bar: 50 µm.

The immunofluorescence studies showed that the keratinocyte hydrogel layer appeared firmly attached to the fibroblast-containing fibrin hydrogel. Both keratinocytes and fibroblasts were viable and grew in their respective layers occupying the entire surface homogeneously (Figure 2).

### 2.5. In Vivo Fabrication of Polymerizable Skin Hydrogel

Both skin hydrogel polymerization and wound healing were analyzed in vivo.

Polymerizable skin hydrogels fabricated with diluted thrombin concentrate were not able to attach to the wound bed. The polymerization time was very slow and no adhesion to the surrounding tissue was observed. The hydrogel was spilled on the back of the mice, not being able to remain in the wound until coagulation was completed.

**Figure 3:**
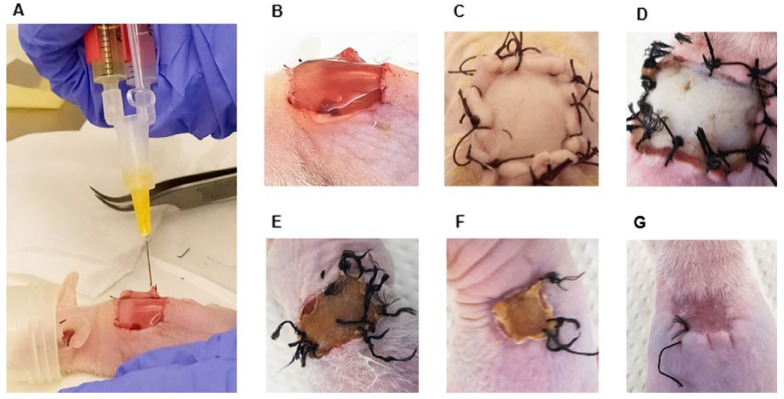
In vivo fabrication of polymerizable skin hydrogel. (**A**) In vivo extrusion of the polymerizable skin hydrogel. (**B**) Extruded hydrogel on the wound bed at room temperature remained stable without leaking. (**C**) Devitalized murine skin sutured to the dorsum of the mouse after surgery. (**D**–**F**) Wound healing follow-up. Devitalized skin shrank and detached gradually from the back of the mouse. (**G**) Regenerated human skin in the back of the athymic mouse. Wound completely healed after 21 days.

In contrast polymerizable skin hydrogel fabricated with thrombin concentrate adhered to the wound bed and its edges, and did not show any detachment before being covered with the devitalized skin in any case. The devitalized skin shrank and spontaneously detached from back of the mouse 14–21 days after transplant. After detachment of the devitalized skin, the regenerated human skin appeared darker and thinner than the surrounding tissue. The skin was completely regenerated, and the wounds were healed and closed in all cases (Figure 3). The control group (hydrogel without human cells) showed no difference with respect to the group treated with the polymerizable skin in terms of wound healing time.

**Figure 4:**
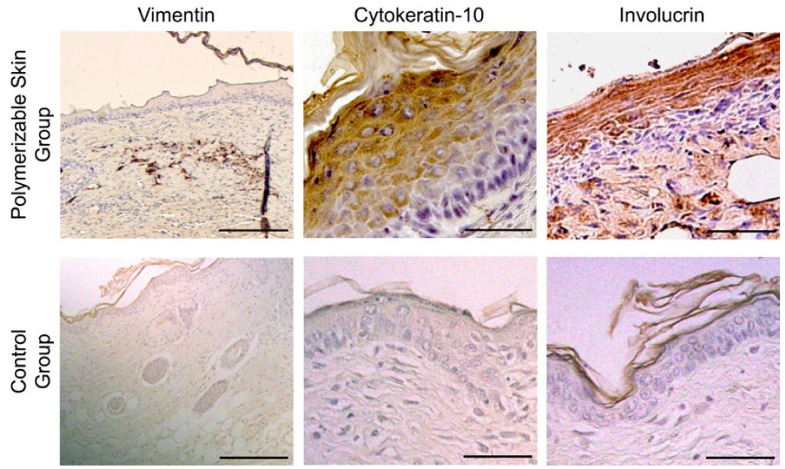
Immunohistochemistry study of the regenerated skin in athymic mouse. 21 days after treatment with the polymerizable skin hydrogel, the human regenerated skin showed positive labelling for involucrin and cytokeratin 10 in the suprabasal layers. Vimentin labelled full thickness of human dermis and some cells located into the dermis of the grafted area. Scale bars: 50 µm. Vimentin scale bar: 200 µm. In the control group (hydrogel without human cells) wound closure is achieved by murine cells from the wound edges. Biopsies from the treated area showed no labelling for the antibodies used.

The immunohistochemical study of the group treated with the polymerizable skin showed the formation of a keratinized epithelium over a dermal layer, which were recognized by human specific anti-involucrin, anti-cytokeratin 10 and anti-vimentin antibodies. Cytokeratin 10 and involucrin were expressed in the spinous, granulous and corneal layers and vimentin was expressed in the human fibroblasts located in the dermis. Immunohistochemical studies of control group (treated with hydrogel without human cells) revealed, as expected, absence of labelling for the aforementioned antibodies (Figure 4).

## 3. Discussion

This study shows the development of a new polymerizable skin hydrogel based on FFP and containing both dermal and epidermal cells. This hydrogel can be applied directly over a wound bed thanks to the rapid polymerization and allows skin regeneration in a full thickness wound model. The polymerizable skin hydrogel described here opens the possibility of a complete autologous system in which all the components (fibroblasts, keratinocytes, fibrinogen and thrombin concentrate) come from the patient themselves.

Clotted plasma is the naturally occurring temporary wound cover involved in efficient re-epithelization and connective-tissue reorganization [17]. Plasma factor I (fibrinogen) is a polymerizable protein, and it represents the clotting faction, while plasma factor IIa (thrombin) is a serine protease that converts soluble fibrinogen into insoluble fibrin strands. Both fibrinogen and thrombin are medical products commonly used in clinical practice. The use of FFP to obtain both components of the hydrogel has the advantage of being cheap, readily available and could prevent the transmission of communicable diseases and immunological reactions if it is obtained from an autologous origin [14,15,16]. Additionally, fibrinogen and thrombin concentrate can be stored with high stability for future use if the patient requires several procedures. These results are consistent with previous studies [26,27,28], where it was proven that coagulation factors are stable when stored at temperatures lower than −20 °C for 24 months without significant changes in most coagulation factors including the fibrinogen and the thrombin, allowing the preservation of this biomaterials for long periods of time.

Neither viability nor cell density was impaired in the presence of fibrinogen or thrombin concentrate. We must recognize that these studies were performed only with dermal fibroblasts because keratinocytes cannot be cultured in the absence of feeder cells, and the latter could invalidate the results by not being able to be distinguished from the keratinocytes. Nevertheless, the fact that the keratinocytes remained in the wound and were able to regenerate human skin in the murine wound healing model suggests that the viability of these cells has not been affected.

In previous models of bioengineered skin based on clotted plasma, coagulation is obtained by recalcification which usually takes between 15–30 min, is temperature dependent (it does not occur at temperatures <37 °C) [13,17,29,30] and constitutes solely the dermal layer. In a secondary step, keratinocytes need to be cultured over the fibroblasts-containing fibrin dermal equivalent, covered with culture medium and cultured for several days prior to its use [13,17]. The polymerizable skin hydrogels described here consist of entrapped fibroblasts and keratinocytes on a fibrin substrate that are clotted using active thrombin concentrate, purified from FFP, in a process that last less than 5 s at room temperature. The fast polymerization of the skin hydrogel allowed it to fix itself to the edges of the wounds without any detachment, which would be impossible using calcium chloride.

The polymerizable skin model was able to support the proliferation of both cell types (fibroblasts and keratinocytes), both in vitro and in vivo. Cells grew and presented their characteristic morphology until the end of the culture as shown by immunohistochemical studies. Dispersedly distributed keratinocytes entrapped within the hydrogel were able to form a continuous cell layer over the dermal component as seen after 11 days of in vitro culture. Even though the level of differentiation was not the same as that observed in the in vivo model, this does not exclude this approach from being capable of providing viable cells to the wound bed, which can promote the correct formation of an epidermis in vivo, as showed after 21 days of maturation in vivo. These findings are consistent with those described by Mazlyzam [31] who demonstrated the formation of a living bilayer human skin equivalent fabricated with entrapped keratinocytes and fibroblasts on independent layers of human fibrin coagulated with calcium chloride.

As well as in previous bioengineered skin models, primary cultures of fibroblasts and keratinocytes must be performed in order to obtain a sufficient number of cells to generate the bioengineered skin or the polymerizable hydrogel. One of the advantages of this new polymerizable skin model is that it is rapidly used since it can be applied without the need for a secondary culture of keratinocytes over the fibroblast containing dermal matrix. In contrast with previous models in which, this secondary cultivation would take at least 10 additional days.

The concentrated thrombin showed such a fast polymerization rate that it was difficult to deposit the polymerizable skin hydrogel homogeneously on the flat, dry transwell surface. By diluting the thrombin concentrate we were able to slow down the polymerization rate to suit our needs. In the in vivo fabrication model, it was decided to use the thrombin concentrate directly to be able to apply the polymerizable skin hydrogel to an irregular, convex and wet wound surface without spilling it. In this case, the polymerizable skin hydrogels were produced by a similar method to the ones that are used to generate fibrin glues, which are characterized by their high adhesion forces [32] and already showed to be of good use in oral and maxillofacial surgeries [33,34], ophthalmological surgeries [35,36] and to prevent bleeding after gastric endoscopic dissection [37].

The regenerated tissue obtained after implantation of the polymerizable skin hydrogel in a full-thickness wound model showed complete maturation and integration in the host tissue despite not being cultured prior to transplantation as evidenced by the immunohistochemistry results. Human fibroblasts are disseminated in the murine dermis, contributing together with recipient’s cells, to dermal regeneration. Epithelial cells were surrounded by the host tissue, and the regenerated epithelium presented the four strata of a normal skin (basal, spinous, granular and corneum strata) as evidenced by the suprabasal layers labelled with cytokeratin 10 and involucrin.

We found no difference in wound healing time between the control group and the polymerizable skin group. This could be attributed to a non-critical wound size, resulting in wound closure due to natural contraction during the healing process. In addition, the plasma hydrogel provides a whole-blood set of cytokines, attachment factors and platelet-derived growth factors that make these plasma-based scaffolds offer a highly proliferative environment for epithelial cells [17] present in the wound edges. Animal models of wound healing do not represent a real clinical situation, since wounds are created de novo and the wound bed is in an optimal situation. Animal models of chronic wounds are very complex to simulate and even more so with athymic animals.

However, in a real clinical situation, a complete healing of the wound would not be achieved with the plasma hydrogel alone. Epithelial stem cells are needed not only to promote wound closure, but also to remain on-site and renew the epithelium throughout the life of the individual. Fibroblasts also play an important role since they collaborate on extracellular matrix and connective tissue formation. Fibroblasts are capable of producing and secreting metalloproteinases, collagen, fibronectin, proteoglycans and several growth factors (VEGF, FGF, PDGF) which are potent angiogenic factors [38].

At the clinical level one of the limitations of our method is that it requires as many cells as the methods previously described [13,17]. This is a major setback in the case of severe burn patients in which a great area of polymerizable skin hydrogel would have to be prepared. However, this will not be as limiting in the case of patients or lesions requiring small areas of polymerizable skin such as: wounds in patients suffering from epidermolysis bullosa, giant congenital nevus resections, chronic ulcers, lesions of the oral cavity, vaginal reconstruction, etc.

One of the advantages of this model is that it does not require adhesion to a solid support for its handling, like our previous model [17,18,19,20,21,39]. This makes it a much simpler model to handle as it can be applied with a conventional syringe.

An additional advantage of this polymerizable skin model for use in a clinical situation is its rapid polymerization. This characteristic can facilitate its application in humid regions with difficult topography such as the oral cavity and the vaginal area. In addition, it may avoid the use of splints and/or prostheses to keep the gel adhered to the wound bed during the wound healing period.

The polymerizable skin hydrogel model here described is based on a cell culture system that has been employed to treat burns for more than 40 years. In addition, FFP derived products used for this work have been shown to be safe for use in humans. For all these reasons, this product is closer to complying with all regulatory requirements in a shorter time frame and thus closer to being transferred to the patient’s bedside.

Having a product, such as the one described in this study, which is easily applied directly into wounds, would result in an improvement in the current treatments for skin defects, not only because it would represent a faster and cheaper alternative to skin equivalents but also because it would ease the overall application of cell products on patients, finally resulting in the improvement of their quality of life.

## 4. Materials and Methods

### 4.1. Thrombin and Fibrinogen Preparation

Blood bags of 220–300 mL fresh frozen plasma (FFP) were obtained from 24 altruistic donors (between 25–65 years of age) from the regional blood and tissue bank (Centro Comunitario de Sangre y Tejidos, CCST, Asturias, Spain) according to the standards of the American Association of Blood Banks and European and Spanish laws and regulations.

FFP was thawed at 37 °C for 20–30 min and 20 mL was retained to use as the clotting component of the hydrogel (hereafter fibrinogen). The rest of the FPP was used to extract the thrombin concentrate by acid precipitation and defibrination with calcium according to the method described by Quarmby J et al. [40] with some modifications. Briefly, 200 mL of FFP was precipitated in 800 mL of 0.04% acetic acid, and the solution was centrifuged at 1500× *g* for 20 min at 21–23 °C. The pellet was resuspended in 20 mL calcium buffer that contained 0.9% sodium chloride, 0.03% sodium bicarbonate and 25 mM calcium chloride, and then the mixture was vortex-mixed for 2 min and incubated at room temperature for 15 min. After incubation, the solution was filtered through a 40 µm nylon cell strainer (Thermo Fisher Scientific, Waltham, MA, USA). The precipitated fibrin retained in the filter was removed, and the thrombin concentrate was collected. The pH of thrombin concentrate in calcium buffer was measured with Fisherbrand™ pH Indicator Paper Sticks (Thermo Fisher Scientific).

Finally, both samples (fibrinogen and thrombin concentrate) were aliquoted and stored at −80 °C until further use.

### 4.2. Fibrinogen Quantification

Fibrinogen concentration was measured using the Clauss method [41], where 100 µL of fibrinogen samples were clotted in the presence of an excess of thrombin (200 µL of Multifibren U^®^ 50 UI/mL, Siemens AG, Munich, Germany) in a Behring Fibrintimer II (Dade Behring, Deerfield, IL, USA) at 37 °C. The clotting times were recorded in duplicate, and the fibrinogen concentration was calculated from a standard curve calculated using the date tables provided by the kit’s manufacturer (Siemens AG).

### 4.3. Thrombin Activity Quantification

The thrombin activity was determined as follows: 100 µL of 2.11 g/L fibrinogen was clotted by adding 200 µL of thrombin concentrate samples diluted with calcium buffer (1:5 to 1:40) in a Behring Fibrintimer II at 37 °C. The clotting times between 18–35 s were recorded in duplicate, and the thrombin activity was calculated from a standard curve set up by clotting 100 µL of 2.11 g/L fibrinogen with different thrombin activities (from 50 to 100 UI/mL).

### 4.4. Stability Study

Effects of freezing and frozen storage of thrombin concentrates and fibrinogen were analyzed by storing them at −80 °C. For this purpose, fibrinogen concentrations (*n* = 6) and thrombin activities (*n* = 6) were measured after 15 and 30 days of frozen storage as previously described.

### 4.5. Primary Cultures of Keratinocytes and Fibroblasts

The present research was conducted according to the guidelines of the Declaration of Helsinki and approved by the Ethics Committee of Principado de Asturias (Protocol Code: 2020.50; 23 March 2020). For this study, three skin biopsies of 2–3 cm^2^ were obtained from deceased organ donors after informed written consent following Spanish laws in organ and tissue donation.

Keratinocytes and fibroblasts were cultured following previously described methods [7] with slight modifications by our laboratory [13,17]. Keratinocytes were cultured in presence of lethally irradiated 3T3 feeder cells. The keratinocyte culture medium was a mixture of Dulbecco’s modified Eagle’s medium (DMEM, Thermo Fisher Scientific) and Ham’s F12 (F12, Thermo Fisher Scientific) (2:1) supplemented with 10% fetal bovine serum (FBS), 100 U/mL penicillin, 0.10 g/L streptomycin, 5.0 µg/mL insulin, 8.33 ng/mL cholera toxin, 0.40 µg/mL hydrocortisone, 1.30 ng/mL triiodothyronine, and 24 µg/mL adenine (Sigma-Aldrich, San Luis, MO, USA). The keratinocyte medium was replaced every three days, and 10 ng/mL epidermal growth factor (EGF) (Sigma-Aldrich) was added since the first replacement. The fibroblast culture medium was a mixture of DMEM and F12 (2:1) supplemented with 10% FBS, 100 U/mL penicillin and 0.10 g/L streptomycin and was replaced every three days. Both cultures were incubated in a humidified 5% CO_2_ atmosphere at 37 °C until confluence (7–12 days). Cultures were amplified to obtain a sufficient number of cells to carry out the experiments (7–12 days).

### 4.6. Viability Assay

Thrombin concentrate and fibrinogen toxicities were studied in fibroblasts by a fluorescence viability test with propidium iodide (red fluorochrome) and Hoechst dye (blue fluorochrome). Dermal fibroblasts were seeded in a 6-well plate (Thermo Fisher Scientific) and cultured until 70–80% confluent. Next, thrombin concentrate and fibrinogen were thawed at 37 °C, and separately diluted in culture medium at 1:1. Then, fibroblasts were incubated with 100 µL/cm^2^ of culture medium (*n* = 4, control group), thrombin concentrate (*n* = 4, thrombin concentrate group) or fibrinogen (*n* = 4, fibrinogen group) for 24 h.

After incubation, cells were washed in PBS and stained with 10 µg/mL propidium iodide (Thermo Fisher Scientific) and 2 µg/mL Hoechst staining (Thermo Fisher Scientific) in DMEM:F12 (2:1) for 30 min at 37 °C. Finally, cells were examined under a DM6000B fluorescence microscope (Leica, Wetzlar, Germany) and photos were taken in triplicate using a DFC310 FX camera (Leica) at ×400 magnification. Next, red and blue nuclei were counted by a single blinded researcher using ImageJ software (National Institutes of Health, Bethesda, MD, USA). To assess toxicity, cell density and cell viability were calculated according to the following formulas.

$$\mathrm{Cell}\;\mathrm{density}\;\left(\mathrm{cells}/{\mathrm{cm}}^{2}\right)=\frac{\mathrm{blue}\;\mathrm{nucleus}\;}{\mathrm{Image}\;\mathrm{area}\;({\mathrm{cm}}^{2})}$$

$$\mathrm{Cell}\;\mathrm{viability}\;\left(\%\right)=\frac{\left(\mathrm{blue}\;\mathrm{nucleus}\;-\;\mathrm{red}\;\mathrm{nucleus}\right)}{\mathrm{blue}\;\mathrm{nucleus}}\times 100$$

### 4.7. Preparation of Polymerizable Hydrogel

The polymerization time of the hydrogel was measured as follows: 150 µL of fibrinogen and 150 µL of thrombin concentrate were mixed in a Behring Fibrintimer II, the clotting times were recorded in duplicate.

Keratinocyte and fibroblast cell cultures were treated with trypsin-EDTA, centrifuged (400× *g*, 10 min) and counted. Keratinocytes were resuspended in 2 mL of fibrinogen at 3 × 10^6^ cells/mL, and then, the fibroblasts were resuspended in 2 mL of fibrinogen at 3.7 × 10^5^ cells/mL. The cell suspensions were drawn into 2 mL syringes (BD Biosciences, CA, USA).

Thrombin concentrate was used at normal concentration and also diluted (1:10) in calcium buffer. To prepare the polymerizable hydrogel, one syringe containing thrombin and one containing fibrinogen/cells (either fibroblasts or keratinocytes) were connected to a single 25 G needle (BD Biosciences), and syringes were simultaneously extruded.

### 4.8. In Vitro Fabrication of Polymerizable Skin Hydrogel

To fabricate the in vitro skin hydrogel, 0.15 mL/cm^2^ of thrombin concentrate, or 0.15 mL/cm^2^ of diluted 1:10 thrombin concentrate, and fibroblast-containing fibrinogen (hereafter dermal layer) were extruded on a 24 mm Corning^®^ Transwell^®^ permeable support (Sigma-Aldrich). The plate was transferred to the incubator to coagulate (5–10 min at 37 °C). Then, 0.05 mL/cm^2^ of thrombin concentrate, or 0.05 mL/cm^2^ of diluted 1:10 thrombin concentrate and keratinocyte-containing fibrinogen (hereafter epidermal layer) was extruded over the dermal layer. The plate was transferred again to the incubator to coagulate (5–10 min at 37 °C). Hydrogels were then covered with keratinocyte culture medium supplemented with 1.5 mg/mL tranexamic acid (B. Braun Medical S.A., Rubí, Spain), and incubated in a humidified 5% CO_2_ atmosphere at 37 °C for 3–4 days. Next, polymerizable skin hydrogels were maintained at the air–liquid interface for 7 days in the keratinocyte culture medium supplemented with 1.5 mg/mL tranexamic acid and 10 ng/mL EGF (Sigma Aldrich, St. Louis, MO, USA), to enhance stratification of the epithelium. Samples were finally fixed in 4% paraformaldehyde for subsequent immunofluorescence analysis.

### 4.9. In Vivo Fabrication and Transplant of Polymerizable Skin Hydrogel

Immunodeficient athymic nude mice (N = 6, male, 6–7 weeks, 25–30 g) were purchased from Charles River Laboratories (Ecully, France) and kept and used at the Animal Experimental Centre of the University of Oviedo (Asturias, Spain) in pathogen-free conditions. All animal experiments were approved by the competent regional authorities (Spanish registration code PROAE 21/2019) and were conducted according to European and Spanish laws and regulations.

Polymerizable skin hydrogel was extruded on the back of each mouse, previously anaesthetized with 2% isoflurane, by the methodology described in the previous section. Briefly, a 4.5 cm^2^ full thickness circular wound was performed on the back of each mouse and the skin was removed and devitalized by three cycles of freezing and thawing (grafting protocol commonly used by our group) [42]. Next, dermal layer and epidermal layer were extruded on the wound bed (polymerizable skin group, *n* = 3), and the polymerizable skin hydrogel was protected from traumas by covering it with the devitalized mouse skin as biological bandage and sutured with a 4-0 nylon suture. A control group (*n* = 3) underwent the same procedure but used only the polymerizable hydrogel, without human skin cells.

Examinations were performed on each mouse on a weekly basis and pictures of the grafted site were taken during the follow-up period. Finally, mice were euthanized by CO_2_ inhalation and tissue samples were harvested and fixed in 4% paraformaldehyde for subsequent immunohistochemical analysis.

### 4.10. Immunofluorescence

In vitro polymerizable skin hydrogels were embedded in paraffin, sectioned and stained with anti-human high molecular weight keratin antibody (1:40, clone 34E12, Dako, CA, USA) and anti-human vimentin (1:100, clone SP20, Abcam, Cambridge, UK). Deparaffinized tissue sections (5 µm) were rinsed with PBS solution twice for 10 min, and permeabilized in a PBS solution containing 0.3% Triton X-100 (Sigma Aldrich) for another 5 min. Following this, the samples were incubated at 4 °C overnight with primary antibodies (1:100) containing 10% normal goat serum (Fischer Scientific, Hampton, NH, USA) as a blocking agent. After several washes, samples were incubated with corresponding secondary antibodies (1:500) (Life Technologies, Carlsbad, CA, USA) for 2 h at room temperature, and were finally stained with 4’,6-diamidino-2-phenylindole (DAPI) to allow nuclei visualization. Photos were captured using the DM6000B fluorescence microscope (Leica) fitted with a DFC310FX camera (Leica).

### 4.11. Immunohistochemistry

The samples from athymic mice’s grafted area (control and polymerizable skin groups) were fixed in 4% paraformaldehyde and embedded in paraffin. Deparaffinized tissue sections (5 µm) were rinsed with PBS solution twice for 10 min, incubated 5 min in 3% hydrogen peroxide (Sigma Aldrich) in absolute methanol (Sigma Aldrich) to inactive endogenous peroxidases, and permeabilized in a PBS solution containing 0.3% Triton X-100 for another 5 min. Following this, the samples were incubated for 48 h at 4 °C with primary antibodies anti-human involucrin (1:100, clone SY5, Sigma Aldrich), anti-human keratin 10 (1:100, clone LH2, Abcam) or anti-human vimentin (1:100, clone SP20, Abcam), containing 10% normal goat serum as a nonspecific reaction blocking agent. Next, samples were incubated with the peroxidase-conjugated secondary antibody (1:200) (Vector Laboratories, Burlingame, CA, USA) for 2 h. Subsequently, the samples were incubated with 0.03% H_2_O_2_ (Vector Laboratories), 0.1% 3,3′-diaminobenzidine (Vector Laboratories) in 0.1 M PBS pH 7.4 for 10–20 s or until a brown precipitate appeared. Finally, nuclei were counterstained with haematoxylin and tissue slides were dehydrated, cleared, and mounted. Photos were captured using the DM6000B bright-field microscope (Leica) fitted with a DFC310FX camera (Leica).

### 4.12. Data Analysis

Graphic representations and linear regression were performed using Excel version 2016 (Microsoft^®^, Redmond, WA, USA), and statistical evaluation was performed using IBM SPSS Statistics version 22 (IBM Corp, Armonk, NY, USA). The normal distribution of tested values was assessed by the Shapiro–Wilk method. Significant differences among defined groups were tested using parametric tests, one-way ANOVA followed by a Student’s *t*-test (for stability study and the cell density in the viability assay) and using the non-parametric test Mann–Whitney U (for the cell viability in the viability assay). A difference at a level of *p* < 0.05 was considered to be statistically significant. The quantitative data is expressed as mean ± SD.

## 5. Conclusions

In conclusion, the polymerizable skin hydrogel described in this work is a novel system based on techniques and products commonly used in tissue engineering. It is a simple, portable and affordable hydrogel that has a demonstrated ability to regenerate skin in a full thickness wound model. Moreover, the methodology and results of this work create the possibility of a completely autologous system in which all the components come from the patient themselves. For all the above-mentioned reasons, we suggest that this hydrogel could be used as an Advance Therapy Medical Product, fulfilling all the pertinent regulations, in a short period of time.

## Figures and Tables

**Table 1:** Viability assay. Fibroblasts viability and cell density after being cultured in the presence of thrombin concentrate and fibrinogen. Samples prepared from FFPs did not show toxicity in fibroblasts since the number of cells per cm^2^ and the cell viability did not decrease significatively.

Group	Cell Viability (%)		Cell Density (Cells/cm^2^)	
Control	98.16 ± 2.03	*p* > 0.05	271 ± 27	*p* > 0.05
Thrombin Concentrate	83.92 ± 21.43		242 ± 29	
Fibrinogen	98.64 ± 1.36		232 ± 20	

## Data Availability

All the obtained data used to support the findings of this study are available from the corresponding author upon reasonable request.
